# A smartphone-based zero-effort method for mitigating epidemic propagation

**DOI:** 10.1186/s13634-023-00984-6

**Published:** 2023-02-01

**Authors:** Qu Wang, Meixia Fu, Jianquan Wang, Lei Sun, Rong Huang, Xianda Li, Zhuqing Jiang

**Affiliations:** 1 School of Automation Science and Electrical Engineering, University of Science and Technology Beijing, Beijing, China; 2 Shunde Innovation School, University of Science and Technology Beijing, Foshan, China; 3 Research Institute of China Unicom, Beijing, China; 4 School of Artificial Intelligence, Beijing University of Posts and Telecommunications, Beijing, China

**Keywords:** COVID-19, Social distance, Contact tracing, Epidemic warning, Human activity recognition, Indoor positioning

## Abstract

A large number of epidemics, including COVID-19 and SARS, quickly swept the world and claimed the precious lives of large numbers of people. Due to the concealment and rapid spread of the virus, it is difficult to track down individuals with mild or asymptomatic symptoms with limited human resources. Building a low-cost and real-time epidemic early warning system to identify individuals who have been in contact with infected individuals and determine whether they need to be quarantined is an effective means to mitigate the spread of the epidemic. In this paper, we propose a smartphone-based zero-effort epidemic warning method for mitigating epidemic propagation. Firstly, we recognize epidemic-related voice activity relevant to epidemics spread by hierarchical attention mechanism and temporal convolutional network. Subsequently, we estimate the social distance between users through sensors built-in smartphone. Furthermore, we combine Wi-Fi network logs and social distance to comprehensively judge whether there is spatiotemporal contact between users and determine the duration of contact. Finally, we estimate infection risk based on epidemic-related vocal activity, social distance, and contact time. We conduct a large number of well-designed experiments in typical scenarios to fully verify the proposed method. The proposed method does not rely on any additional infrastructure and historical training data, which is conducive to integration with epidemic prevention and control systems and large-scale applications.

## Introduction

**Fig. 1:**
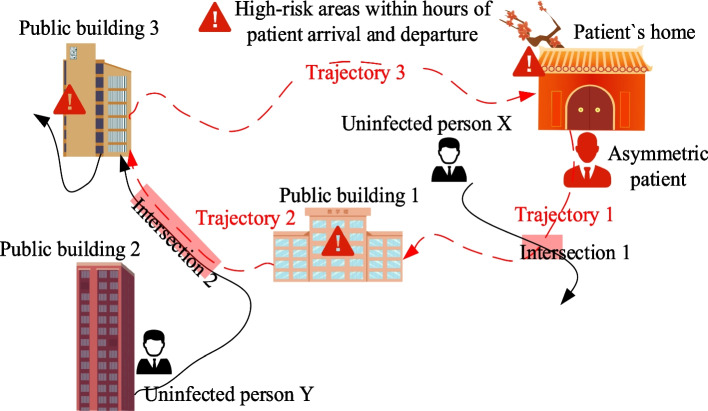
The history of the infected person and all the people who met or were infected

At the end of 2019, the new type of coronavirus pneumonia (COVID-19) caused by the SARS-COV-2 virus broke out in Wuhan and spread rapidly around the world, bringing huge impacts and challenges to the global medical and economic system. As of December 21, 2022, a total of 654,420,532 cases of new coronary pneumonia have been diagnosed worldwide, and a total of 6,624,023 cases have died [1]. Prevention and control measures such as rapid isolation of cases and strict restrictions on the movement and contact of people have effectively cut off the spread of the virus and have made important contributions to blocking the spread of COVID-19 [2]. As shown in Fig. 1, contact tracking aims to identify risk regions, and trace contacts and spreaders, which is an effective strategy to control epidemic spread [3, 4]. Initially, the infected person was tracked by manual contact. Although effective, as the number of infected people grows, this manual contact tracking method has exposed many shortcomings (expensive manpower and material resources, and tracking personnel are vulnerable to virus infection). On the other hand, COVID-19 has an incubation period of up to 14 days, and it is difficult to control the spread of the virus simply by quarantining the sick. It is difficult to track down individuals with mild or asymptomatic symptoms with limited human resources.

The academic community has successively tried several solutions to identify infected persons, track contacts, and remind users to maintain social distancing. At present, domestic and foreign has developed based on pseudolites, radio frequency identification, ultra-wideband, inertial sensors, wireless local area network, ultrasonic, visible light, magnetic field, Bluetooth, computer vision and other technologies to achieve sub-meter to ten-meter accuracy of personnel positioning and tracking system [5]. At present, domestic and international solutions for close contact tracking and social distance reminding of infectious disease epidemics still have poor positioning and tracking accuracy, relying on additional positioning base stations and historical training data, requiring professional knowledge such as medical care, and failing to remind safe social distance in real time and many other shortcomings, and it is challenging to promote and apply on a large scale.

The smartphone that users carry with them has built-in GPS, accelerometer, gyroscope, sound, and other rich sensors and has strong computing and storage capabilities. It is an ideal epidemic tracking and early warning platform. This article is based on the built-in sensors of mobile smartphones to identify users’ talking, sneezing, coughing and other activities that are closely related to the spread of the epidemic, and real-time positioning and tracking of user trajectories, and building an epidemic early warning system to remind users to maintain social distance. The proposed method has the advantages of not relying on additional infrastructure, historical training data, and no need for professional knowledge. The key contributions of our study are as follows:

- We propose a zero-effort epidemic warning method based on epidemic-related voice activity recognition and autonomous positioning. This method comprehensively evaluates the infection risk from three aspects: epidemic-related vocal activity, social distance, and contact time.
- We propose an integrated data and knowledge-driven activity recognition method based hierarchical attention mechanism and temporal convolutional network to recognize voice activity relevant to epidemics spread. The proposed method reduces the number of convolutional layers and expands the receptive field by integrating hierarchical attention mechanisms and fully mines data dependencies to improve recognition accuracy.
- We propose a social distance estimation method based on pedestrian dead reckoning (PDR) using smartphones carried by pedestrians. This method does not rely on any additional infrastructure and historical training data, which is conducive to integration with epidemic prevention and control systems and large-scale application.
- We propose a contact time estimation method that combines Wi-Fi network logs and social distance to comprehensively judge whether there is spatiotemporal contact between users and determine the duration of contact.

The following sections of this paper are organized as follows: Sect. 2 reviews the previous related works. Section 3 details the proposed smartphone-based zero-effort epidemic warning method. Section 5 thoroughly evaluates the proposed method in typical scenarios. Finally, Sect. 5 draws a conclusion and outlines our future work.

## Related work

Due to the rapid spread of COVID-19, how to assess the infection risk has become a current research hotspot [6]. We review the previous related works of human voice activity recognition, social distance estimation, and contact tracing.

### Voice activity recognition

Voice activity recognition has a wide range of applications, such as human–computer interaction, health monitoring, activity understanding, scene recognition, and smart home control. Plenty of studies on voice activity recognition have been developed. The key issue of recognizing different voice activities is to extract effective features from the acoustical signal that may contain noise. These features are mainly classified into time domain and frequency domain. The common time-domain features are periodicity, zero-crossing rate, short-time energy, loudness, and sharpness [7]. Spectral flatness, frequency component, high/low-frequency rate, Mel-frequency cepstral coefficient (MFCC), and Log Mel Filter-bank are the most used frequency-domain features. In addition, various classification techniques such as clustering [8], *k*-nearest neighbor [9], support vector machine, fuzzy-rule [10], Gaussian mixture models [11], random forest, linear discriminant analysis, logistic regression, decision trees are used for voice activity recognition. Sensor noise is the main reason affecting recognition accuracy. In recent years, deep learning has been widely used to solve numerous sensor noise problems. Lee et al. [12] proposed a spectral–temporal attention-based voice activity recognition method. Kim et al. [13] utilized an adversarial domain adaptation technique to perform robust voice activity recognition out of noisy background signals. To capture the entire temporal information of voice signals, Zhang et al. [14] stacked a global temporal pooling layer on multiple local temporal pooling layers. Although many voice activity recognition methods have been presented, there are still deficiencies in recognition accuracy, robustness, processing rate, or computation overhead in practical applications.

### Social distance estimation

Social distancing is a public health measure aimed at preventing close contact between infected person and healthy person during an infectious disease outbreak, to reduce the chance of disease spreading. Many technologies, such as positioning technology, wireless communication, artificial intelligence, and big data, have been developed to remind and urge people to maintain social distance [15]. Particularly the positioning systems effectively remind users to maintain a safe distance by measuring the distance between users and notifying them automatically if they are too close to each other [15].

Many wireless positioning technologies, such as GNSS, Cellular, Wi-Fi, RFID, UWB, and Bluetooth, are adopted to enable social distancing. Rajasekar [16] utilized cost-effective RFID tags and a smartphone as an RFID reader to identify social distance. Alsaeedy et al. [17] leveraged cellular networks to detect social distance. Cunha et al. [18] developed a wearable social distance monitoring system that leverages the received signal strength indication (RSSI) of the Wi-Fi signals emitted by devices carried by other users to estimates the proximity distance between the users. Lam and She [19] estimated social distance based on the received signal strength of the BLE beacon. To prevent the spread of COVID-19, Kobayashi et al. [20] constructed a social distance monitoring system that periodically sends and receives Bluetooth messages to students on the university campus to sense the distance between users. MySD [15] leveraged the BLE and GPS signal to estimate the distance between people. Abdulqader et al. [21] and Zheng et al. [22] utilized ultrasonic sensor to estimate the distance between users. Bian and colleagues [23] developed a social distance monitoring system based on oscillating magnetic field to monitor the social distances between users. However, these social distance estimation methods rely on additional infrastructure, and their application is limited.

On the other hand, several social distance monitoring systems based on fixed or mobile digital cameras have been developed. Yeshasvi et al. [24] designed an effective social distancing estimation and alerting system that utilizes surveillance video as input to estimate humans’ social distance and urge person to maintain social distance. Ahmed et al. [25] leveraged the object recognition method based on YOLO v3 to recognize pedestrians and estimate their mutual distance. To real-time monitor the social distance in low-light environments, Rahim et al. [26] proposed an efficient solution based on YOLO v4 and fixed ToF camera. Al-Khazraji et al. [27] developed an intelligent monitoring physical distances system that not only senses the physical distance in real time, but also offers timely feedback to users who do not observe the social distance. Bashir et al. [28] designed a cost-effective Internet of Things system to monitor physical distances and body temperatures using the Caffe model in OpenCV. Neelavathy et al. [29] proposed a Bluetooth and camera-based smart social distance monitoring application that predict the social distances between two persons using deep learning and image processing techniques. However, the video-based method has the following three limitations. First, this method relies on additional video surveillance equipment (e.g., camera). Second, surveillance video is easily affected by light, which means that this method cannot work effectively at night or in dark environments. Third, this method cannot interact with the smartphone carried by the user and cannot provide the user with real-time risk warnings.

### Contact tracing

Contact tracing aims to track users who have encountered an infected person [30]. Contact tracing has been recognized by the World Health Organization (WHO) as the most effective epidemic control measure [31]. Recognizing the importance of contact tracing, many studies on contact tracing systems have been developed [32]. Some commercial solutions conduct contact tracing with GPS [33], RFID [34], ultra-wideband [35], BLE [36–38], Wi-Fi [31, 39], cellular [40–42], vision [43], and other technologies. To sense mobile social interactions, Banerjee et al. [44] proposed virtual compass, which effectively perceives the mutual distance between users, but cannot obtain direction information. Guo et al. [45] and Rezaei et al. [46] proposed an automatic infection risk assessment method that utilizes captured surveillance videos to identify potentially infected person by droplet-transmitted model.

At the national level, many countries have developed contact tracking systems. China designed a health code system [47] based on QR codes. The system pushes warning messages to users who are too close to the infected person [48]. South Korea detects the proximity to the infected person using the GPS data from smartphone carried by users [49]. Canada designed a COVID-19 exposure notification APP named COVID Alert [50] to track pedestrian movement trajectories and push a notification to the pedestrian who possibly exposes to the coronavirus. Australia designed COVIDSafe [51] that leverages BLE signal to detect the proximity between persons. The United Nations Technology Innovation Laboratory (UNTIL) has developed a new social distance application called 1ponit5 based on Bluetooth. Switzerland designed SwissCovid APP [51] that detects the proximity utilizing the BLE signal on the smartphone. Singapore designed TraceTogether [52] that utilizes Bluetooth to discover and locally record clients in close proximity to a user. However, Bluetooth-based contact tracing solutions are vulnerable to response attacks [53]. Different from TraceTogether, the UK designed Google/Apple Contact Tracing system called NHS COVID-19 [54] that does not record the user’s real identity. In SwissCovid, a decentralized privacy protection protocol is utilized to protect user identity. Italy designed Immuni [55], which is a contact tracing application based on BLE signal and privacy-preserving method. Apple and Google have jointly developed an epidemic tracking tool ‘contact tracing’ [56] to help users determine whether they are in close contacts of patients with new coronary pneumonia. They have proved themselves as powerful tools, helping human beings to control the epidemic situation, but many of them are found to have problems of low efficiency and high cost [32].

## Materials and methods

**Fig. 2:**
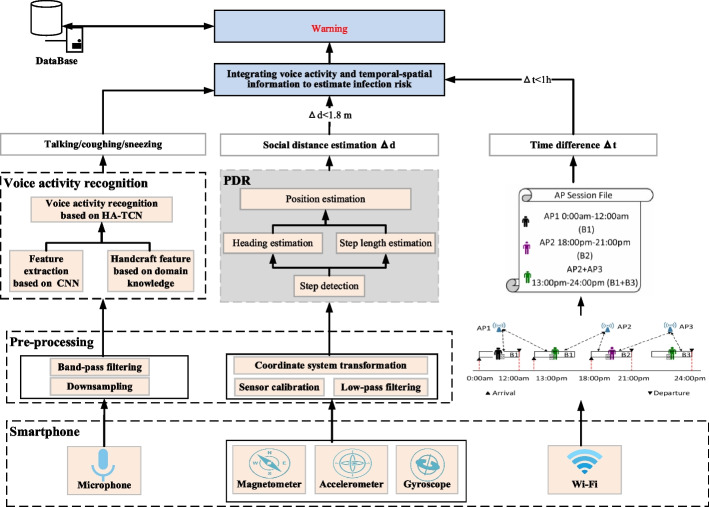
The system architecture of the proposed epidemic warning method based on epidemic-related voice activity recognition and spatiotemporal information

As shown in Fig. 2, we recognize epidemic-related voice activities by hierarchical attention mechanism and temporal convolutional network. Subsequently, we estimate the social distance between users through smartphone. Furthermore, we need to combine Wi-Fi network logs and social distance to comprehensively judge whether there is spatiotemporal contact between users and determine the duration of contact. Finally, we estimate infection risk based on epidemic-related voice activities, social distance, and contact time.

### Integrated data and knowledge-driven method for epidemic-related voice activity recognition

When infected individual talks, coughs, or sneezes, the droplets are sprayed from the mouth or nose into the air. These fine droplets may be inhaled by others. Droplets containing pathogens become the main medium for virus transmission. In this paper, we recognize human vocal activity, especially sneezes and coughs, through the microphone built-in smartphone. As shown in Fig. 2, we propose an integrated data and knowledge-driven voice activity recognition method based on time series deep learning, which converts sound signals into time–frequency series and uses hierarchical attention-based temporal convolutional network (HA-TCN) as the basis to recognize speaking, sneezing, coughing, and other voice activity that is closely related to the spread of epidemics.

The method is mainly composed of three parts: sound signal preprocessing, sound wave feature extraction, and classification model. The sound signal preprocessing part takes the sound wave data collected by the mobile phone microphone as input and uses the short-term logarithmic energy to accurately intercept the effective sound wave signal after noise reduction by the band-pass filter; the sound wave feature extraction part is used to extract and process the domain knowledge feature of the acoustic signal further improves the accuracy of the classification model. The classification model part takes the preprocessed effective acoustic signal and domain knowledge features as input, extracts the hidden feature representation in the input from the encoder layer, and then inputs it to the HA-TCN, and finally represents the feature through the linear layer Converted into activity classification results as output.

#### Preprocessing and feature extraction

The pronunciation of the experimenter, the acquisition equipment, and the surrounding environment will affect the quality of the audio signal, resulting in the occurrence of mute, aliasing, noise, distortion, and other phenomena. Due to the presence of environmental noise in the original sound wave signals collected by smartphones, it is often impossible to obtain good classification results by directly inputting the raw sound data collected by the mobile phone microphone into the neural network for classification tasks. Therefore, preprocessing the collected sound signals is a necessary means to obtain good classification results. This paper uses band-pass filtering to eliminate environmental noises, so as to effectively improve the signal-to-interference plus noise ratio (SINR) of the collected acoustic signals.

Manual segmentation cannot accurately find the start and end of the sound. The purpose of endpoint detection is to remove the silent part and finally get effective sound content. This paper uses the double-threshold algorithm of short-term energy and the short-term average zero-crossing rate for voice endpoint detection. The algorithm can accurately determine the start and end positions of the effective signal in the sound sample and separate the effective sound signal from the ambient noise.

Sensor feature extraction is a critical step in recognizing activity patterns. To accelerate the speed of model convergence and effectively improve model classification accuracy, we extract time-domain and frequency-domain features based on the acoustic domain knowledge.

In the short-term energy calculation, we use the hamming window with a length of ${S}_{1}$ to subframe the acoustic signal $x\left(t\right)$ collected by the microphone and then use Eq. (1) to calculate the short-term logarithmic energy of each frame, and the calculation equation for the average logarithmic short-term energy $STE_{p} \left( j \right)$ of the *j*th frame is shown in Eq. (2).

1 $$E\left( i \right) = 10{\text{log}}\left( {\mathop {\mathop \sum \limits_{n = 0} }\limits^{{S_{i} - 1}} x_{i} \left( n \right)^{2} } \right)$$

2 $$STE_{p} \left( j \right) = \left\{ {\begin{array}{*{20}l} {\left( {1 - \alpha } \right)STE_{P} \left( {j - 1} \right) + \frac{\alpha }{{P_{n} }}\mathop \sum \limits_{i = 1}^{{P_{n} }} E\left( i \right),} \hfill & {j > 1} \hfill \\ {\frac{1}{{P_{n} }}\mathop \sum \limits_{i = 1}^{{P_{n} }} E\left( i \right),} \hfill & {j = 1} \hfill \\ \end{array} } \right.$$

where ${P}_{n}$ is the length of each frame, and $\alpha$ is a constant.

In addition to short-term energy features, we also choose energy entropy features as the model input. The energy entropy feature mainly describes the distribution of the sound signal in the time domain and reflects the continuity of the sound wave signal. The energy entropy feature is computed as follows:

3 $${H}_{i}=-\sum_{k=1}^{K}{e}_{j}{\mathrm{log}}_{2}\left({e}_{j}\right)$$

4 $${e}_{j}=\frac{{E}_{subFram{e}_{j}}}{{E}_{fram{e}_{i}}}$$

5 $${E}_{fram{e}_{i}}=\stackrel{K}{\sum_{k=1}}{E}_{subFram{e}_{k}}$$

where *K* is the number of the subframe; ${e}_{j}$ represents the ratio of the total energy of the *j*th subframe to the total energy of a frame in the entire signal frame; ${E}_{fram{e}_{i}}$ is the total energy of the i-th frame signal; and ${E}_{subFram{e}_{j}}$ is the energy of the *j*th subframe.

Short-time zero-crossing rate ${Z}_{n}$ indicates the number of times the signal amplitude passes through the zero point in each frame of signal, reflecting the frequency characteristics of the frame signal. The short-time zero-crossing rate ${Z}_{n}$ of the *i*th frame signal is as follows,

6 $${Z}_{n}=\frac{1}{2}\sum_{m=0}^{N-1}\left|sign\left[{x}_{n}\left(m\right)\right]-sign\left[{x}_{n}\left(m-1\right)\right]\right|$$

where the sign(*x*) function represents the sample position of the zero point in the signal segment *x*.

The spectrum centroid reflects the main concentrated area of the spectrum energy in the frequency band. The smaller the value of the spectrum centroid, the more spectrum energy is concentrated in the low-frequency range. The spectral centroid of the *i*th frame signal is as follows,

7 $${C}_{i }=\frac{\stackrel{{L}_{n}}{\sum_{k=1}}k{X}_{i}\left(k\right)}{\stackrel{{L}_{n}}{\sum_{k=1}}k{X}_{i}\left(k\right)}$$

where ${X}_{k}$ is the *k*th spectral line of the *i*th frame signal and *f* is the signal length of one frame.

The spectrum extension mainly describes the distribution of the acoustic signal around the centroid of its spectrum.

8 $${S}_{i}=\sqrt{\frac{\stackrel{{L}_{n}}{\sum_{k=1}}{\left(k-{C}_{i}\right)}^{2}{X}_{i}\left(k\right)}{\stackrel{{L}_{n}}{\sum_{k=1}}{X}_{i}\left(k\right)}}$$

Spectral entropy reflects the uniformity of the acoustic signal in the frequency domain.

9 $${H}_{i}=-\stackrel{K}{\sum_{k=1}}{n}_{k}{\mathrm{log}}_{2}\left({n}_{f}\right)$$

10 $${n}_{k}=\frac{{E}_{k}}{\stackrel{K}{\sum_{l=1}}{E}_{l}}$$

The spectrum flux represents the change of the spectrum between two adjacent frames. It is equivalent to calculating the sum of squares of the difference between the two frames of the spectrum after normalization. The calculation equation is:

11 $$F{l}_{\left(i,i-1\right)}=\stackrel{{f}_{n}}{\sum_{k=1}}{\left(E{N}_{i}\left(k\right)-E{N}_{i-1}\left(k\right)\right)}^{2}$$

The short-term power spectral density is a time–frequency characteristic that reflects the strength of each frequency band in the period corresponding to each frame. It can simultaneously reflect the time-domain and frequency-domain characteristics of reflected acoustic wave signals at different positions and is very suitable for analyzing time-varying and non-static reflected acoustic wave signals.

12 $$psd=\frac{y\times {y}^{*}}{N}$$

13 $$y=fft\left(signal,N\right)$$

#### Hierarchical attention-based temporal convolutional network for epidemic-related voice activity recognition

In the HA-TCN architecture, the convolution window between each hidden layer increases layer by layer. This dilated convolution structure can make each hidden layer consistent with the size of the input sequence. The dilated convolution structure allows the model to obtain a sufficiently large receptive field with only a shallow layer.

**Fig. 3:**
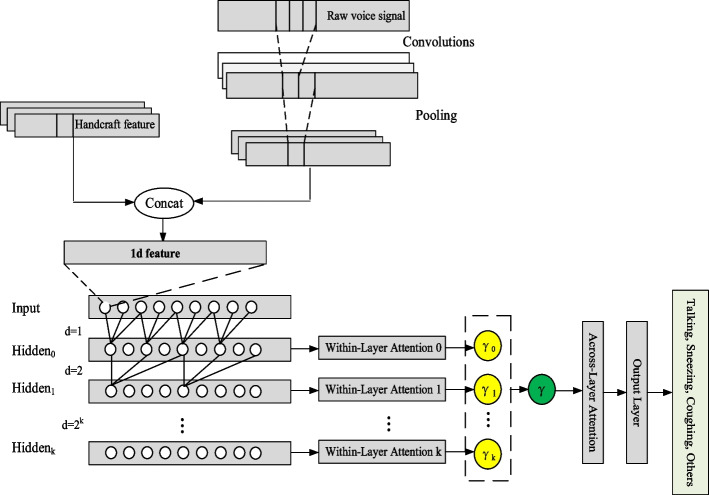
Architecture of hierarchical attention-based temporal convolutional network

In this paper, the time–frequency sequence feature output by the preprocessing module is used as the input of the HA-TCN model. As shown in Fig. 3, for each time step, a one-dimensional time–frequency sequence is first extracted through a sub-network composed of Ns convolutional layers, and the network output is a one-dimensional feature representation. After the spatial feature extraction is completed, the one-dimensional feature is used as input through the main network composed of three layers of Temporal Block. Each layer of the Temporal Block includes a CNN layer, a causal convolution layer, an optional dropout layer and batch normalization (Batch Normalization) layer, and uses ReLU as the activation function. The expansion coefficient d of the cavity convolution between different layers is set to 1, 2, or 4 exponential growths according to the depth increase of the number of layers. The convolution kernel size between each convolution layer is 2 × 1. In the TCN model, the dilated convolution operation $F$ on elements end with index $s$ of the sequence $X$ is defined as:

14 $$F\left(s\right)=\left(X{*}_{d}f\right)\left(s\right)=\stackrel{k-1}{\underset{i=0}{\sum f\left(i\right)\cdot {X}_{s-d\cdot i}}}$$

where $k$ is the convolution kernel size, $d$ is the dilation factor, and each $(s-d\cdot i)$ is the index of an element from the ‘past’ part in the input $x$.

The entire end-to-end classification model takes a 40 × 3 × 1 time–frequency feature vector as input and outputs a 4 × 1 classification result.

15 $$Output\left(i\right)=TCN\left({X}_{i}\right)$$

To reduce the convolutional layers required to expand the receptive field, dilated convolutions are used in TCN. In the convolution kernel of dilated convolution, there is a certain gap between adjacent nodes, which allows dilated convolution to obtain a broader range of information without changing the convolution kernel size. The receptive field size is expressed as:

16 $$R{F}_{L}=1+(k-1)\cdot {\sum }_{i=0}^{L-1}{d}_{i}$$

where ${d}_{i}$ is the dilation factor of the *i*th layer causal convolution. When using dilated convolution, let ${d}_{i}={b}^{i}$ to make the receptive field grow exponentially with the depth of the network. $b$ is the expansion coefficient.

Residual blocks [57] help to solve the gradient instability problem and are widely used in deep networks. In the residual block, the output of the multi-layer network $f$ is added to the original input $x$ and output through the activation function $G$.

17 $$y=G(x+f(x))$$

**Fig. 4:**
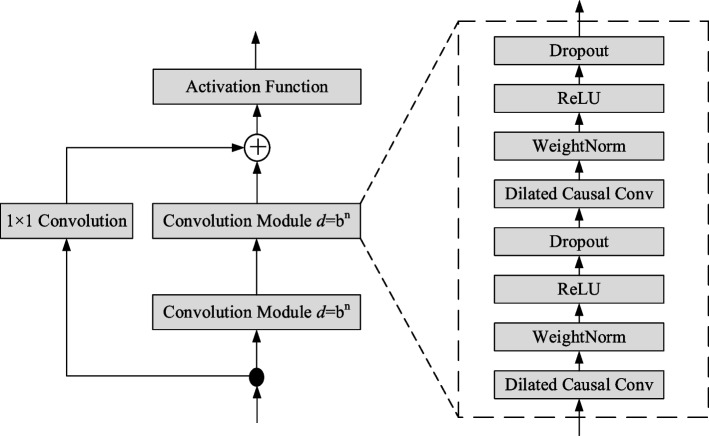
Residual unit in the TCN

As shown in Fig. 4, the residual block of TCN contains two convolution modules. Each convolution module consists of dilated causal convolution, weight normalization, activation function, and dropout. ${H}^{(i)}=\{{h}_{0}^{(i)},{h}_{1}^{(i)},\cdots ,{h}_{T}^{(i)}\}$ and ${H}^{(i+1)}=\{{h}_{0}^{(i+1)},{h}_{1}^{(i+1)},\cdots ,{h}_{T}^{(i+1)}\}$ are the outputs of the *i*th and $i+1$th residual blocks in TCN, respectively. In the residual block, the dilation factor of the two-layer causal convolution remains unchanged. If the dimensions of the original input and that of the convolutional layer output are different, the addition operation can be performed after dimension transformation by 1 × 1 convolution.

The attention mechanism [58] is a simulation of the attention of the human brain. The attention mechanism highlights vital features and improves model performance by weighing different features. It has been widely used in machine translation and computer vision. We utilize a hierarchical attention mechanism across network layers [59] to refine the temporal dependencies and extract significant features. The HA-TCN contains $K$ hidden layers. The within-layer attention weight ${\alpha }_{i}$ is calculated as follows:

18 $${\alpha }_{i}=\mathrm{softmax}(\mathrm{tanh}({w}_{i}^{T}{H}_{i}))$$

19 $${H}_{i}=[{h}_{0}^{i},{h}_{1}^{i},\dots ,{h}_{T}^{i}]$$

where ${H}_{i}$ is the matrix consisting of convolutional activations at layer $i$, $i={0,1},\dots ,K$; ${w}_{i}$ is a trained parameter vector; and $(\cdot {)}^{T}$ denotes the transpose operation.

The combination ${\gamma }_{i}$ of convolutional activations for layer $i$ is calculated as:

20 $${\gamma }_{i}=ReLU({H}_{i}{\alpha }_{i}^{T})$$

After executing each within-layer attention layer, the convolutional activations are transformed as follows:

21 $$M=[{\gamma }_{0},{\gamma }_{1},\dots ,{\gamma }_{i},\dots ,{\gamma }_{K}]$$

Similarly, the across-layer attention layer takes $M$ as the input to calculate the final sequence representation used for classification:

22 $$\alpha =\mathrm{softmax}(\mathrm{tanh}({w}^{T}M))$$

23 $$\gamma =ReLU(M{\alpha }^{T})$$

### Social distance estimation based on pedestrian dead reckoning

Close contact provides conditions for droplet transmission. When people talk, droplets are ejected from the mouth. These fine droplets may be inhaled by others. Droplets containing pathogens become the main medium for virus transmission. Therefore, effective estimation of social distance is crucial to determine whether a person is highly likely to be infected during social interaction activities.

According to the characteristics of pedestrians’ periodic motions, pedestrian dead reckoning uses inertial sensor data to identify step events and estimate step lengths and uses a magnetometer to estimate pedestrian heading, thereby realizing position estimation. Step detection, step length estimation, and heading estimation are closely linked and affect each other. The step detection result is used for sensor data segmentation. The accuracy and real-time performance of step detection directly determine the accuracy of heading and step length estimation. PDR calculates the pedestrian position $({x}_{1},{y}_{1})$ at moment ${t}_{1}$ based on the inertial movement distance ${d}_{0}$, inertial heading ${\theta }_{0},$ and initial position $({x}_{0},{y}_{0})$.

24 $$\left\{\begin{array}{l}{x}_{1}={x}_{0}+{l}_{0}\mathrm{cos}{\theta }_{0}\\ {y}_{1}={y}_{0}+{l}_{0}\mathrm{sin}{\theta }_{0}\end{array}\right.$$

Likewise, pedestrian position $({x}_{2},{y}_{2})$ at moment ${t}_{2}$ is calculated (with distance, heading, and last position) as follows:

25 $$\left\{\begin{array}{l}{x}_{2}={x}_{1}+{l}_{1}\mathrm{cos}{\theta }_{1}={x}_{0}+{l}_{0}\mathrm{cos}{\theta }_{0}+{l}_{1}\mathrm{cos}{\theta }_{1}\\ {y}_{2}={y}_{1}+{l}_{1}\mathrm{sin}{\theta }_{1}={y}_{0}+{l}_{0}\mathrm{sin}{\theta }_{0}+{l}_{1}\mathrm{sin}{\theta }_{1}\end{array}\right.$$

More generally, pedestrian position $({x}_{k},{y}_{k})$ at moment ${t}_{k}$ is calculated as follows:

26 $$\left\{\begin{array}{l}{x}_{k}={x}_{0}+{\sum }_{i=0}^{k-1}{l}_{i}\mathrm{cos}{\theta }_{i}\\ {y}_{k}={y}_{0}+{\sum }_{i=0}^{k-1}{l}_{i}\mathrm{sin}{\theta }_{i}\end{array}\right.$$

where ${\theta }_{i}$ and ${l}_{i}$ are pedestrian heading and movement distance from ${t}_{i-1}$ to ${t}_{i}$, respectively.

#### Magnetic-aided step detection

**Fig. 5:**
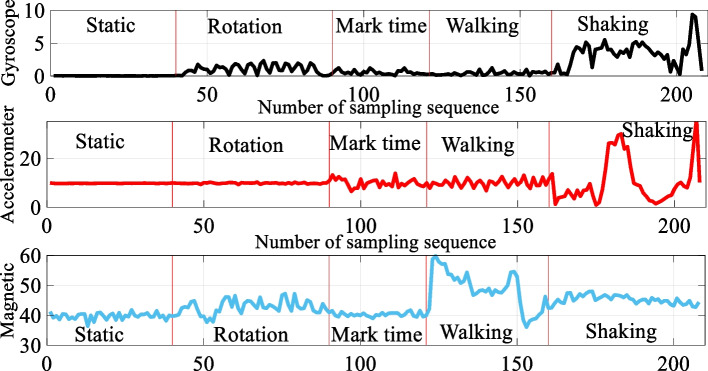
Acceleration, gyroscope, and magnetic signal changes under pedestrian complex walking modes

Step detection is the basis of PDR algorithms. As shown in Fig. 5, considering complex pedestrian activities, such as shaking smartphone phone or rotating smartphone caused by actions such as calling, texting, and playing games, a large error will be generated for the traditional step detection method based on the acceleration modulus threshold. Based on the characteristics that the intensity of the geomagnetic signal changes less at the same location and greatly changes at different locations, this paper applies magnetic data to pedestrian step detection to improve the accuracy and robustness of step detection.

This paper utilizes the sliding window mechanism to analyze and count the acceleration, gyroscope, and magnetic data and calculate the mean value of the acceleration and variance of the gyroscope and magnetic data in the sliding window. When the acceleration mean value, gyroscope and magnetic variance are greater than each predetermined threshold. It is determined that the pedestrian has a new step.

27 $${\text{if}}\;\left( {(a_{m} > A_{th} ) \cap (\delta_{g} > G_{\delta } ) \cap (\delta_{m} > M_{\delta } )} \right)$$

where

28 $$\left\{\begin{array}{l}{a}_{m}=\frac{1}{N}{\sum }_{t=1}^{N}{a}_{t}\\ {\delta }_{g}=\sqrt{\frac{1}{N}{\sum }_{t=1}^{N}{({g}_{t}-\frac{1}{N}{\sum }_{t=1}^{N}{g}_{t})}^{2}}\\ {\delta }_{m}=\sqrt{\frac{1}{N}{\sum }_{t=1}^{N}{({m}_{t}-\frac{1}{N}{\sum }_{t=1}^{N}{m}_{t})}^{2}}\end{array}\right.$$

where the acceleration mean threshold ${A}_{th}$; the gyro variance threshold ${G}_{\delta }$; and the magnetic data variance threshold ${M}_{\delta }$ are obtained by experiments.

#### Adaptive step length estimation

Traditional step length estimation methods cannot adapt to the dynamic changes of pedestrian walking patterns. Considering that pedestrian step length is related to walking speed, step frequency, and other factors, this paper constructs a binary linear step length model based on step frequency and exercise intensity as follows.

29 $${L}_{i}=\alpha\cdot { f}_{i}+\beta \cdot {\delta }_{i}^{a}+\gamma$$

30 $${f}_{i}={1}/ {{(t}_{k}-{t}_{k-1})}$$

31 $${\delta }_{i}^{a}=\sqrt{\frac{{\sum }_{i=1}^{n}{({a}_{i}-\overline{a })}^{2}}{n}}$$

where *α* and *β* are the linear regression coefficients of step frequency ${f}_{i}$ and acceleration variance ${\delta }_{i}^{a}$; $\gamma$ is constant; and ${a}_{k}$ is the *k*th acceleration amplitude in the sliding window.

#### Robust heading estimation based on multi-source fusion

The gyroscope can only estimate the amount of attitude change but cannot give absolute heading information, and the attitude estimation error continues to accumulate over time. Although the heading estimation based on magnetic sensors can give an absolute heading estimate, it is susceptible to the surrounding ferromagnetic materials and other electromagnetic interference in a complex indoor environment, leading to deviations in the heading estimation. The fusion of the heading given by the gyroscope and magnetic data effectively enhances the accuracy and robustness of heading estimation.

The heading angle based on the magnetic field is calculated as follows.

32 $$\left\{\begin{array}{ll}\Psi =180-\left(\mathrm{arctan}\left(\frac{{m}_{hy}}{{m}_{hx}}\right)*\frac{180}{\pi }\right)& {m}_{hx}<0\\\Psi =-\left(\mathrm{arctan}\left(\frac{{m}_{hy}}{{m}_{hx}}\right)*\frac{180}{\pi }\right)& {m}_{hx}>0,{m}_{hy}<0\\ \begin{array}{l}\Psi =360-\left(\mathrm{arctan}\left(\frac{{m}_{hy}}{{m}_{hx}}\right)*\frac{180}{\pi }\right)\\\Psi =90\\\Psi =270\end{array}& \begin{array}{l}{ m}_{hx}>0,{m}_{hy}>0\\ {m}_{hx}=0,{m}_{hy}<0\\ {m}_{hx}=0,{m}_{hy}>0\end{array}\end{array}\right.$$

where ${m}_{hx}$ and ${m}_{hy}$ are the projections of the magnetism on the horizontal plane of the local navigation coordinate system, respectively.

33 $$\left\{\begin{array}{l}{m}_{hx}={m}_{x}^{c}cos\left(\Phi \right)+{m}_{y}^{c}sin\left(\theta \right)\mathrm{sin}\left(\Phi \right)-{m}_{z}^{c}cos\left(\theta \right)sin(\Phi )\\ {m}_{hy}={m}_{y}^{c}cos\left(\theta \right)+{m}_{z}^{c}sin(\theta )\end{array}\right.$$

where ${m}_{x}^{c}$, ${m}_{y}^{c}$ and ${m}_{z}^{c},$ are the magnetic observations on the *X*-, *Y*-, and *Z*-axes in the carrier coordinate system; the roll angle $\theta$ and pitch angle $\Phi$ are directly obtained by the Android API.

The heading estimation based on magnetic field provides initial heading information for the heading estimation based on the gyroscope. Using the correlation between the heading of magnetic field and the heading of gyroscope can not only effectively eliminate the heading estimation error caused by indoor magnetic interference, but also calibrate the accumulated error of the gyroscope. This paper uses the following fusion strategy to perform a weighted fusion of the previous heading, magnetic heading, and gyroscope heading to obtain accurate and robust heading estimation ${\Psi }_{k}$ of current step.

34 $$\left\{\begin{array}{ll}{\Psi }_{{k}}={{\alpha }}_{1}{\Psi }_{{k}-1}+{\beta }_{1}{\Psi }_{{m},{k}}+{\gamma }_{1}{\Psi }_{{g},{k}}& {\Psi }_{\Delta ,{c}}\le {\Psi }_{\tau ,{c}}, {\Psi }_{\Delta ,{m}}\le {\Psi }_{\tau ,{m}}\\ {\Psi }_{{k}}={{\alpha }}_{2}{\Psi }_{{k}-1}+{\beta }_{2}{\Psi }_{{m},{k}}+{\gamma }_{2}{\Psi }_{{g},{k}}& {\Psi }_{\Delta ,{c}}\le {\Psi }_{\tau ,{c}},{\Psi }_{\Delta ,{m}}>{\Psi }_{\tau ,{m}}\\ \begin{array}{l}{\Psi }_{{k}}={{\alpha }}_{3}{\Psi }_{{k}-1}+{\beta }_{3}{\Psi }_{{m},{k}}+{\gamma }_{3}{\Psi }_{{g},{k}}\\ {\Psi }_{{k}}={{\alpha }}_{4}{\Psi }_{{k}-1}+{\beta }_{4}{\Psi }_{{m},{k}}+{\gamma }_{4}{\Psi }_{{g},{k}}\\ \begin{array}{l}{\Psi }_{{k}}={{\alpha }}_{5}{\Psi }_{{k}-1}+{\beta }_{5}{\Psi }_{{m},{k}}+{\gamma }_{5}{\Psi }_{{g},{k}}\\ {\Psi }_{{k}}={\Psi }_{{g},{k}}\end{array}\end{array}& \begin{array}{l} {\Psi }_{\Delta ,{c}}>{\Psi }_{\tau ,{c}},{\Psi }_{\Delta ,{m}}\le {\Psi }_{\tau ,{m}},{\Psi }_{\Delta ,{g}}<{\Psi }_{\tau ,{g}}\\ {\Psi }_{\Delta ,{c}}>{\Psi }_{\tau ,{c}},{\Psi }_{\Delta ,{m}}\le {\Psi }_{\tau ,{m}},{\Psi }_{\Delta ,{g}}\ge {\Psi }_{\tau ,{g}}\\ \begin{array}{l}{\Psi }_{\Delta ,{c}}>{\Psi }_{\tau ,{c}},{\Psi }_{\Delta ,{m}}>{\Psi }_{\tau ,{m}},{\Psi }_{\Delta ,{g}}<{\Psi }_{\tau ,{g}}\\ {\Psi }_{\Delta ,{m}}>{\Psi }_{\tau ,{m}}, {\Psi }_{\Delta ,{g}}\ge {\Psi }_{\tau ,{g}}\end{array}\end{array}\end{array}\right.$$

where ${\Psi }_{k}$ represents the current step heading; ${\Psi }_{k-1}$ represents the previous step heading; ${\Psi }_{m,k}$ represents the magnetic-based heading of current step; ${\Psi }_{g,k}$ represents the gyroscope-based heading of current step; ${\Psi }_{\Delta ,c}$ is the absolute value of the difference between ${\Psi }_{m,k}$ and ${\Psi }_{g,k}$; ${\Psi }_{\Delta ,m}$ is the absolute value of the difference between ${\Psi }_{m,k}$ and ${\Psi }_{m,k-1}$; ${\Psi }_{\Delta ,g}$ is the absolute value of the difference between ${\Psi }_{g,k}$ and ${\Psi }_{g,k-1}$; ${\Psi }_{\tau ,c}$, ${\Psi }_{\tau ,m}$ and ${\Psi }_{\tau ,g}$ are the thresholds of ${\Psi }_{\Delta ,c}$, ${\Psi }_{\Delta ,m}$, and ${\Psi }_{\Delta ,g}$, respectively; these three threshold parameters are obtained based on experiments; ${\alpha }_{i}$,${\beta }_{i},$ and ${\gamma }_{i}$ $(i=1,2,3,4,5)$ are the weights of ${\Psi }_{k-1}$, ${\Psi }_{m,k},$ and ${\Psi }_{g,k}$, respectively. These three parameters are also obtained based on experiments.

### Contact time estimation based on Wi-Fi network logs and social distance

**Fig. 6:**
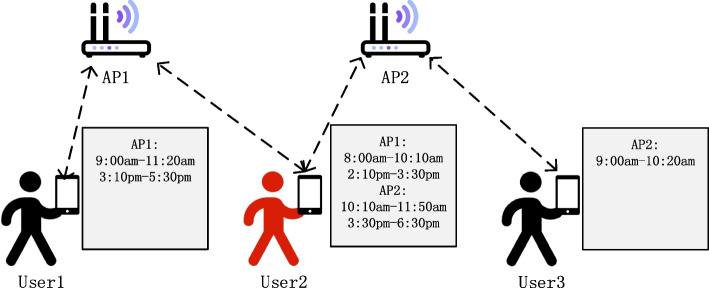
Wi-Fi network logs

The smartphones carried by pedestrians are usually turned on and connected to Wi-Fi. Therefore, the Wi-Fi network log is an effective way to judge the intersection of time. Figure 6 indicates an example of Wi-Fi log information. From the figure, we can find that user 1 connects to Wi-Fi access point 1(AP1), during the 9:00 a.m.–11:20 a.m. period and 3:10 p.m.–5:30 p.m. period, with the association duration is 280 min. According to WHO’s COVID-19 guidelines [60], close contact is defined as two people staying within 1 m for 15 min or more. User 1 and User 2 are simultaneously connected to Ap1 for 90 min. User 2 was connected to Ap2 at the same time as User 3 for 10 min. Therefore, we preliminarily conclude that there is temporal contact between user 1 and user 2 (duration > 15 min). We also preliminarily conclude that there is no temporal contact between user 2 and user 3 (duration ≤ 15 min). However, Wi-Fi network logs cannot accurately reflect the physical distance between users. Therefore, we need to combine Wi-Fi network logs and social distance to comprehensively judge whether there is spatiotemporal contact between users and determine the duration of contact.

### Infection risk estimation

**Fig. 7:**
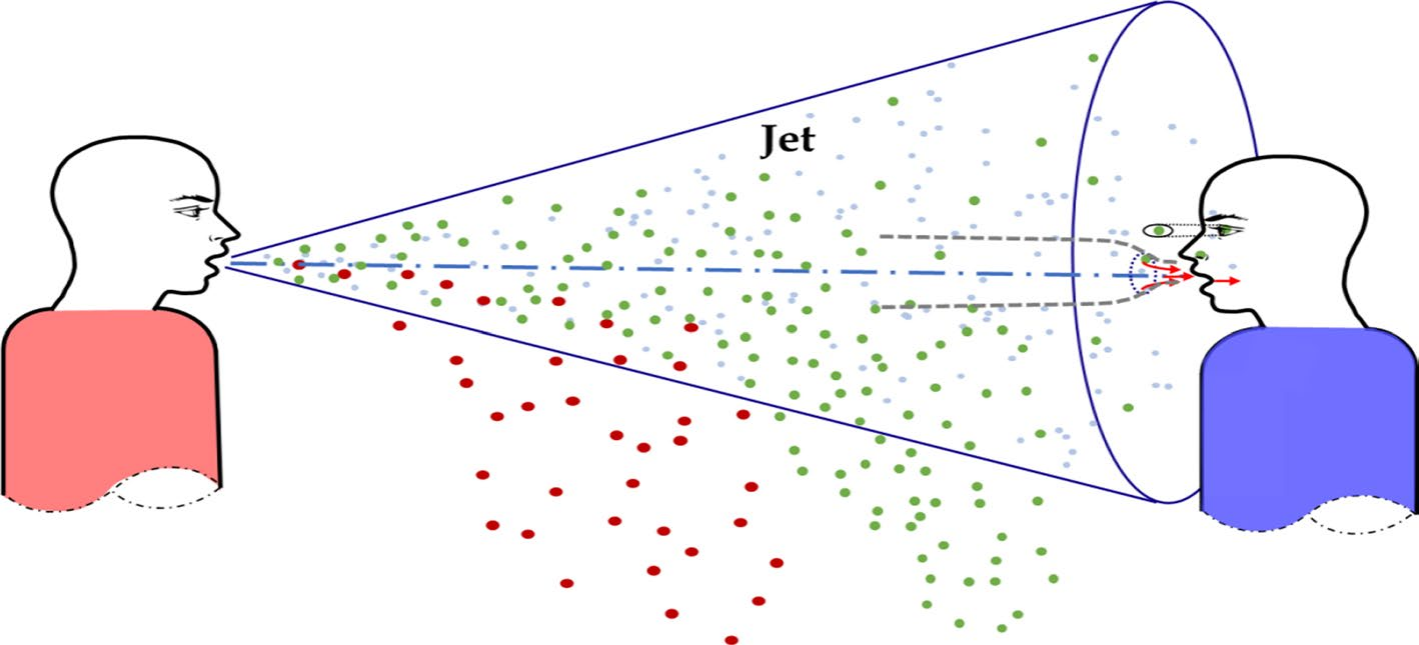
Turbulent jet model

When an infected person talks, coughs, or sneezes, the virus is sprayed into the air along with droplets from the mouth or nose. According to [61], the respiratory airflow of an infected person can be modeled as a turbulent jet model, as shown in Fig. 7. The turbulent jet model consists of a large droplet route and a short-range airborne route. The left person is identified as the infection source, and the other is identified as the target (susceptible). The large droplets are deposited directly on the facial membranes (eyes, nostrils, and mouth) of susceptible persons, while short-range airborne is directly inhaled by the mouth. When the droplet is larger than 100 microns, the spray distance of speaking is less than 0.2 m, and the spray distance of coughing is less than 0.5 m. The short-range airborne route usually predominates. The smaller the exhaled droplets, the farther they travel [61]. Direct face-to-face contact of a susceptible person with a source is the most dangerous situation.

**Fig. 8:**
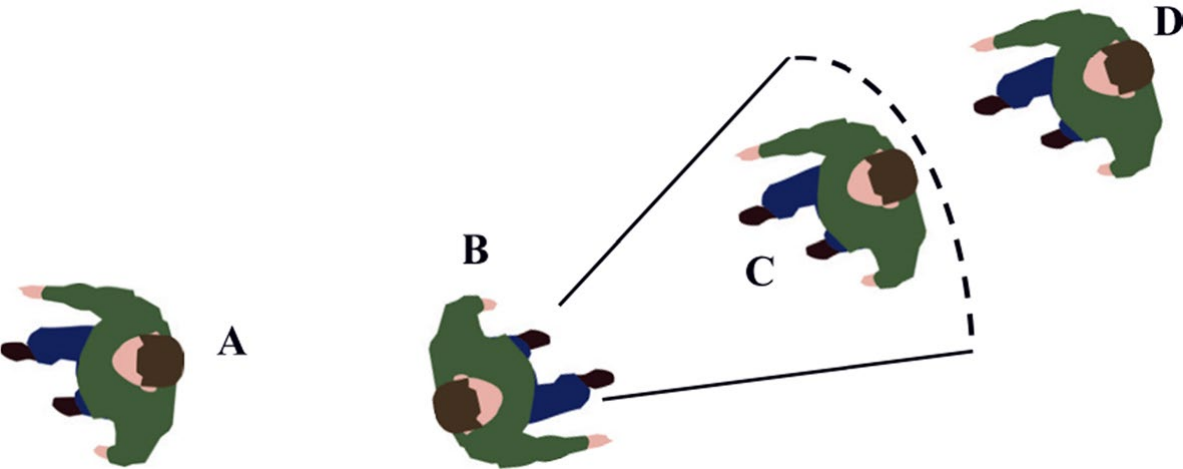
Effect of orientation on the risk of infection

According to the turbulent jet model, orientation is a key factor in determining the infection risk. As shown in Fig. 8, pedestrians B and C are talking face to face. If B is a virus carrier, then the probability of C being infected is extremely high. Although pedestrian A is very close to virus carrier B, the probability of A being infected is low. This is because A and B are in a back-to-back relationship. Since pedestrian D keeps a safe distance from virus carrier B, the probability of D being infected is low.

**Fig. 9:**
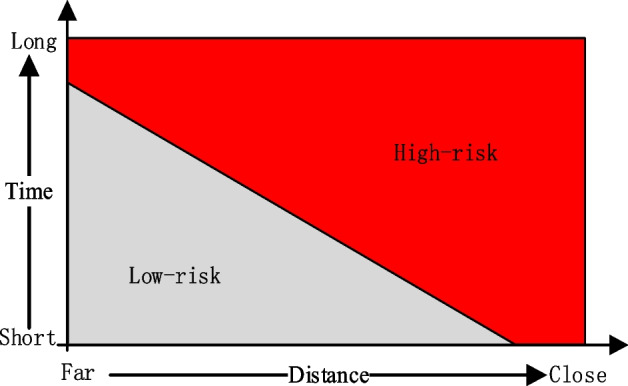
Infection risk versus distance and time

In addition to voice activity, contact distance and time are also critical factors in determining infection risk. In terms of the distance and duration of interaction between the user and the infected person, the possible infection risk is shown in Fig. 9. Although the possibility of infection is greater when the user is in close contact with an infected person, the infection risk is relatively low if the user is in close contact with the infected person for less than 1 s. On the other hand, if a user spends an extended period with an infected person, even if they maintain enough social distance from each other, the risk of exposure is high. Even if the distance from an infected person is greater than the safe threshold, close contact with an infected person or being with an infected person in a confined space for a long time is considered a high infection risk.

## Experimentation and evaluation

In this section, we fully evaluate the proposed method. The performance measures and experimental setup are first described. Section 4.2 verifies the performance of the epidemic-related voice activity recognition method. Section 4.3 verifies the performance of the social distance estimation method based on pedestrian dead reckoning.

### Performance measures and experimental setup

Epidemic-related voice activity recognition is a typical multi-classification problem. We use the confusion matrix (CM), accuracy, precision, recall, and weighted *F*-measure (${F}_{w}$) as classification metrics to evaluate the actual performance of the proposed epidemic-related voice activity recognition method in this paper. The calculation of these indicators can be represented by Eqs. (35)–(38).

35 $$Accuracy=\frac{TP+TN}{TP+TN+FP+FN}$$

36 $$Precision=\frac{TP}{TP+TN}$$

37 $$Recall=\frac{TP}{TP+FN}$$

where $TP$, $TN$, $FP,$ and $FN$ represent the number of true positives, true negatives, false positives, and false negatives, respectively.

Due to the class imbalance problem, we consider the proportion of samples to the *F*1 score by weighting. This evaluation metric is called the weighted *F*-measure (${F}_{w}$).

38 $${F}_{w}=\sum_{i}2\times {\omega }_{i}\times \frac{{Precision}_{i}\cdot {Recall}_{i}}{{Precision}_{i}+{Recall}_{i}}$$

where $i$ is the class index, and ${w}_{i}=\frac{{n}_{i}}{N}$ with samples’ number of *i*th class ${n}_{i}$, the total number of samples $N$.

**Fig. 10:**
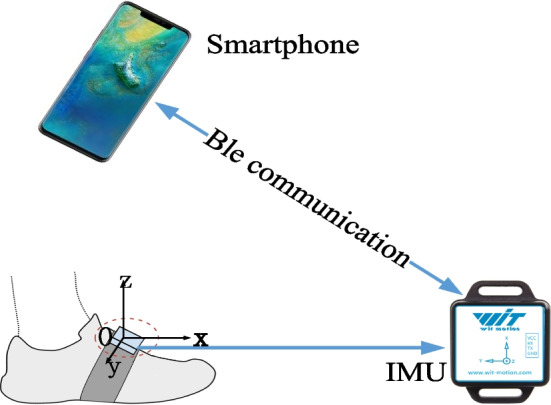
The devices used in experiments

A foot-mounted inertial navigation system (INS) provides high-frequency positioning results and controls the positioning error within 0.3% of the total traveled distance [62]. Therefore, we construct the localization performance evaluation system, as shown in Fig. 10, to evaluate the proposed method. The evaluation system consists of an Android smartphone and a foot-mounted INS module. The precise pedestrian position from the foot-mounted INS module is sent to the smartphone via Bluetooth low energy (Ble) and synchronizes with the measurements of smartphone-embedded MEMS sensors. We use the final position error over total traveled distance (ε/TTD), step detection rate (SDR), step length error (SLE), and circular error probability (CEP) as metrics to quantify the performance of the proposed positioning method.

To verify the localization accuracy and robustness of the proposed method, we invited a group of heterogeneous volunteers with different body shapes to evaluate the proposed method. The experiment was conducted by four males and three females, ranging from 18 to 45 years old. The data collection devices included six smartphones of different brands. Tables 1 and 2 provide a detailed explanation of the subjects and devices.

**Table 1:** Description of volunteers

Volunteers	Gender	Age	Height (cm)	Weight (kg)
V1	M	28	174	74
V2	M	27	164	67
V3	F	24	165	48
V4	M	36	192	81
V5	F	26	171	52
V6	F	28	158	49
V7	M	32	165	73

**Table 2:** Description of devices

Devices	Phone	OS Version	Inertial sensor
D1	Huawei Mate 20	Harmony 10.0	Icm20690
D2	Huawei P9	Android 7.0	LSM330
D3	Honor V30 pro	Harmony 2.0	Lsm6dsm
D4	Samsung Galaxy S6	Android 6.0	MPU6500
D5	OnePlus 8T	Android 11.0	Bmi26x
D6	Mi 9 pro	MIUI 12.5.1	Lsm6ds3c

### Epidemic-related voice activity recognition in typical scenarios

**Fig. 11:**
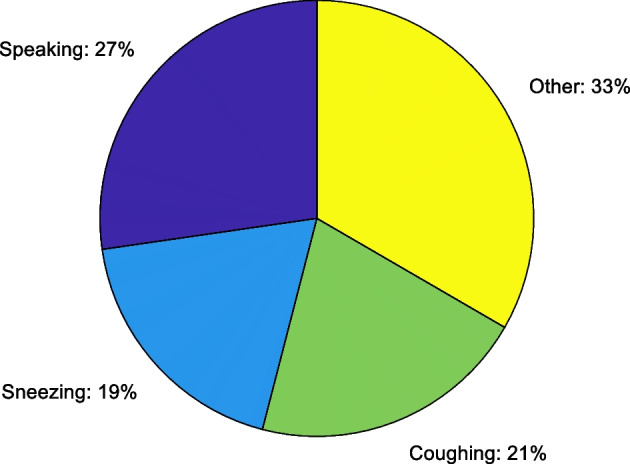
The distribution of samples

**Fig. 12:**
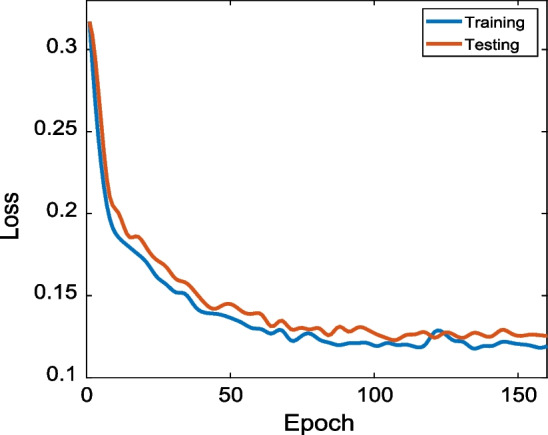
Loss curve

We collected voice samples in rooms, offices, corridors, metro, and outdoor and shopping malls to verify the classification performance of the epidemic-related voice activity recognition model. Smartphones sample the voice data at a frequency of 44,100 Hz. The distributions for the four voice activities are shown in Fig. 11. We randomly divided the collected samples into training (70%) and testing (30%) sets. To train the HA-TCN-based voice recognition method, we use the RMSprop algorithm to optimize and update network parameters. If the learning rate is not set to an appropriate value after several epochs, the loss value tends to change little or no longer. To solve this problem, we adopt a learning rate decay strategy. After every 15 epochs, the learning rate is set to 0.1 times the original value, which can make the loss continue to decrease and reach a very low value. To prevent overfitting, we adopt a dynamic stopping criterion for model training. When the loss function value does not decrease within 50 epochs, the system automatically stops iterations. The loss curve of the training and testing sets is presented in Fig. 12. It can be seen from Fig. 12 that when the number of epochs is less than 70, the loss value decreases faster. After 70 epochs, the loss value changes little. Finally, it stabilizes below 0.14, indicating that the robustness of the model is strong. In this work, we train the epidemic-related vocal activity recognition model on the PC with python language and PyTorch deep learning platform and transfer the trained model to smartphone side to recognize activity recognition model, which is a low-overhead process that can meet real-time requirements.

**Fig. 13:**
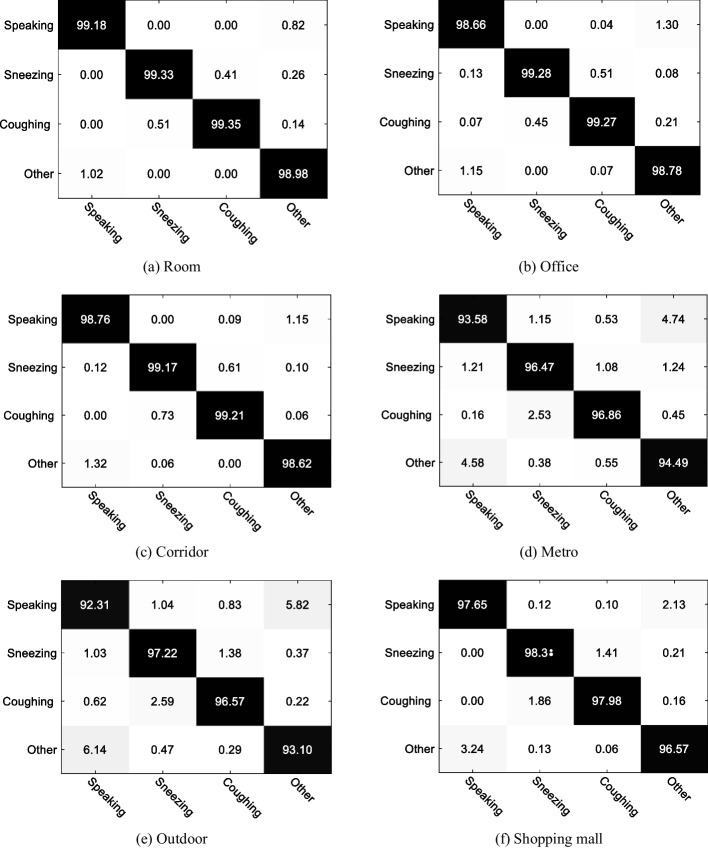
Epidemic-related voice activity recognition results in typical scenarios

The experimental results are shown in Fig. 13 and Table 3. The scene with the lowest recognition accuracy is metro and shopping mall, which are 95.35% and 94.79%, respectively; from the confusion matrix in Fig. 13, it can be seen that speaking is easily misjudged as other voice, and other voice is easily misjudged as speaking. From an audio point of view, metro and shopping malls are noisy and contain some announcements, causing confusion. The scene with the highest classification accuracy is room, which is 99.2%, which is relatively closed and lacks noise. As shown in Table 3, the recognition accuracy of room, office, corridor, metro, outdoor, and shopping mall is 99.20%, 98.99%, 98.94%, 95.35%, 94.79%, and 98.58%, respectively. The average recognition accuracy of six scenes is 97.64%.

**Table 3:** Epidemic-related voice activity recognition results in typical scenarios

Scenarios	Accuracy (%)	Precision (%)	Recall (%)	Fw score (%)
Room	99.20	98.89	99.18	99.03
Office	98.99	98.97	98.96	98.97
Corridor	98.94	98.78	98.98	98.88
Metro	95.35	94.96	96.72	95.83
Shopping mall	94.79	95.16	95.853	95.51
Outdoor	98.58	98.41	98.64	98.52
Average	97.64	97.50	98.06	97.77

We also compare the proposed method with CNN, LSTM, and TCN-based activity recognition methods. We compare the four methods in terms of accuracy, precision, recall, and Fw Score. The experimental results are shown in Table 4. TCN uses the depth of the network to store historical information and simultaneously adds dilated convolutions to replace the input gate, forget gate, and output gate in the recurrent neural network. Compared with LSTM and CNN, TCN can better extract effective information while reducing parameters and enhancing model performance. Compared with TCN, the HA-TCN method reduces the number of convolutional layers and expands the receptive field by integrating hierarchical attention mechanisms and thoroughly mines data dependencies to improve recognition accuracy. As shown in Table 4, the proposed method has an accuracy rate of more than 97.64% for recognizing epidemic-related voice activities, which is significantly better than other compared methods.

**Table 4:** Comparison with other methods

Methods	Accuracy (%)	Precision (%)	Recall (%)	Fw score (%)
CNN	93.96	93.96	92.57	93.26
LSTM	95.20	95.21	95.20	95.17
TCN	96.79	96.77	96.46	96.61
HA-TCN	97.64	97.50	98.06	97.77

### Positioning accuracy in typical scenarios

**Fig. 14:**
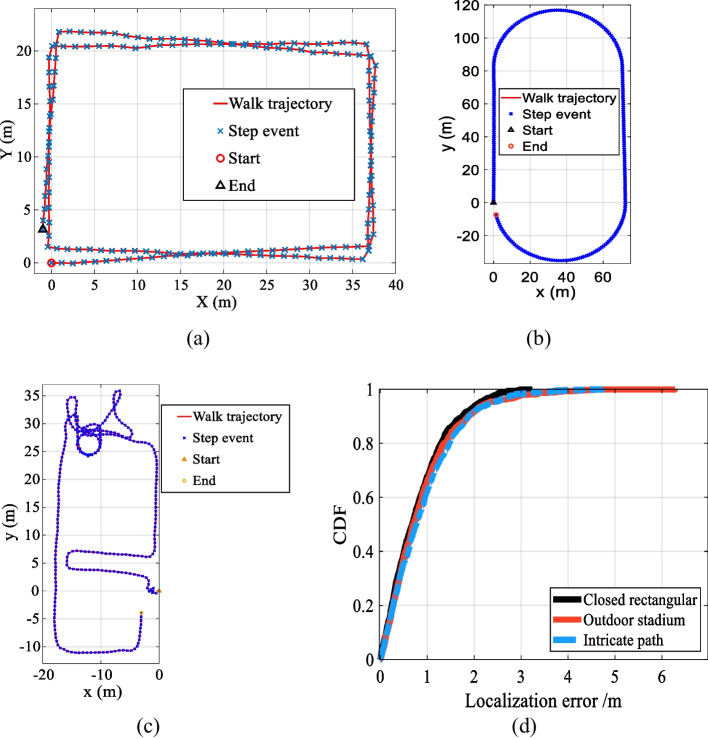
Walking trajectories and CDF in three typical scenarios. **a** Rectangular. **b** Stadium. **c** Intricate path. **d** CDF of three scenarios

To evaluate the proposed social distance estimation method, we conduct well-designed and extensive experiments in three typical navigation scenarios: rectangular (Walk 100 m in a reinforced concrete office building), stadium (Take a walk around the outdoor stadium), and intricate path (Walk 210 m casually in a reinforced concrete office building). We invite multiple volunteers with noticeable physical differences to conduct multiple experiments along the planned path using heterogeneous equipment. Figure 14 shows some walking estimated trajectories and the cumulative distribution function (CDF) of the proposed method. In addition to step detection accuracy and step length estimation error, we also count the circular error probability (CEP) by calculating the distances between the estimated and actual positions. As shown in Table 5, the statistical results show that the SDR, SLE, CEP (50%), CEP (75%), and CEP (95%) of the proposed method are 99.35%, 4.4 cm, 0.71 m, 1.23 m, and 2.93 m, respectively. The ε/TTD of closed rectangular, outdoor stadium, and intricate path is 1.57%, 1.94%, and 2.38%, respectively. The localization performance of the three scenarios is very similar, which proves that the proposed method has satisfactory universality and robustness.

**Table 5:** Positioning results of three typical scenarios

Scenarios	Length (m)	SDR (%)	SLE (cm)	CEP 50% (m)	CEP 75% (m)	CEP 95% (m)	*ε*/TTD (%)
Rectangular	228	99.39	4.4	0.71	1.16	2.11	1.57
Stadium	400	99.53	4.3	0.69	1.28	4.21	1.94
Intricate path	210	99.13	4.6	0.73	1.26	2.48	2.38
Mean	279.33	99.35	4.4	0.71	1.23	2.93	1.96

To justify the superiority of the proposed method, we compared the proposed method with the following PDR methods.

- Traditional PDR leverages step detection based on acceleration zero-crossing, fixed step length, and the heading from Android’s compass to reckon pedestrian locations.
- SmartPDR [63] detects step events, estimates step length with a three-axis accelerometer, and determines heading direction with a three-axis magnetometer and a three-axis gyroscope.

**Fig. 15:**
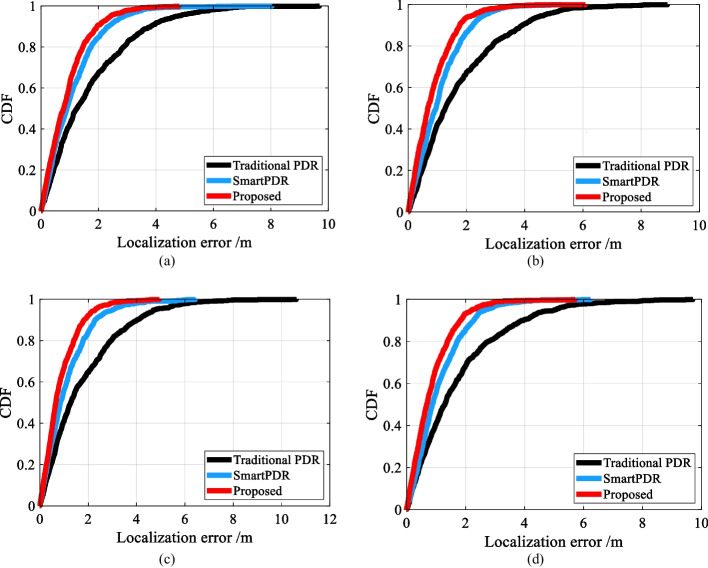
Comparative experiments in four typical scenarios. **a** Office. **b** Metro station. **c** Shopping mall. **d** Outdoor stadium

Many factors, such as different devices, different pedestrians, different walking patterns, and different terminal attitudes, etc., will affect positioning accuracy. To make a fair comparison, we build an offline dataset containing four typical positioning scenarios of office, metro station, shopping mall, and outdoor stadium and compare the proposed method and above compared methods on the same offline dataset to evaluate the positioning accuracy of different methods. The experimental results are shown in Table 6 and Fig. 15. As shown in Table 6, thanks to the assistance of magnetic field information, the step detection accuracy of the proposed method significantly outperforms that of the traditional PDR and SmartPDR. Adaptive step length estimation accuracy is significantly better than fixed threshold-based step length estimation accuracy. ε/TTD of the proposed method is 2.03%, while those of Traditional PDR and SmartPDR are 4.60% and 2.46%, respectively. Figure 15 shows the cumulative error distribution of different methods. As shown in Fig. 15, the red line of the proposed method is steeper than the other plots, indicating that our proposed method’s overall error is significantly lower than those of the compared methods.

**Table 6:** Comparative experiments

Methods	SDR (%)	SLE (cm)	CEP 50% (m)	CEP 75% (m)	CEP 95% (m)	*ε*/TTD (%)
Traditional PDR	93.29	6.8	1.30	2.41	5.01	4.60
SmartPDR	98.83	4.8	0.87	1.56	2.68	2.46
Proposed	99.13	4.6	0.69	1.23	2.21	2.03

## Conclusion

Keeping controlled activities and safe social distancing (at least 6 feet) is an effective non-pharmacological approach for limiting epidemic spread. In this paper, we propose a zero-effort epidemic warning method based on epidemic-related voice activity recognition and autonomous positioning using smartphones carried by pedestrians. The proposed method does not rely on any additional infrastructure and historical training data, which is conducive to integration with epidemic prevention and control systems and large-scale applications. We conduct many experiments in typical scenarios to verify the performance of epidemic-related voice activity recognition and social distance estimation methods. Due to the lack of real epidemic transmission data, it is difficult to complete the infection risk assessment experiment in this paper. In future work, we seek to cooperate with the epidemic prevention and control department to improve the proposed warning system. In addition, user privacy is an issue that must be considered in future research.

## Data Availability

Data sharing is not applicable to this article.

## Abbreviations

AP: Access point
CDF: Cumulative distribution function
CEP: Circular error probability
CM: Confusion matrix
HA-TCN: Hierarchical attention-based temporal convolutional network
INS: Inertial navigation system
HA-TCN: Hierarchical attention-based temporal convolutional network
MFCC: Mel-frequency cepstral coefficient
PDR: Pedestrian dead reckoning
RSSI: Received signal strength indication
SDR: Step detection rate
SINR: Signal-to-interference plus noise ratio
SLE: Step length error
TCN: Temporal convolutional network
TTD: Total traveled distance
WHO: World health organization
